# Depletion of OLFM4 gene inhibits cell growth and increases sensitization to hydrogen peroxide and tumor necrosis factor-alpha induced-apoptosis in gastric cancer cells

**DOI:** 10.1186/1423-0127-19-38

**Published:** 2012-04-03

**Authors:** Rui-hua Liu, Mei-hua Yang, Hua Xiang, Li-ming Bao, Hua-an Yang, Li-wen Yue, Xue Jiang, Na Ang, Li-ya Wu, Yi Huang

**Affiliations:** 1 Center for Clinical Molecular Medicine; Ministry of Education Key Laboratory of Child Development and Disorders; Key Laboratory of Pediatrics in Chongqing; Chongqing International Science and Technology Cooperation Center for Child Development and Disorders; Children's Hospital, Chongqing Medical University, Chongqing 400014, China; 2 Ministry of Education Key Laboratory of Laboratory Medical Diagnosis, Chongqing Medical University, Chongqing 400016, China; 3 Department of Neurosurgery, Xinqiao Hospital, Third Military Medical University, Chongqing 400037, China; 4 Division of Human Genetics, Cincinnati Children's Hospital Medical Center, Cincinnati 45229, USA; 5 Department of Urologic Surgery, Yubei Pepole's Hospital, Chongqing 401120, China; 6 Department of Health Care, Nanjing Maternity and Child Health Care Hospital, Nanjing 210004, China; 7 Department of Biochemistry and Molecular Biology, Chongqing Medical University, Chongqing 400037, China

**Keywords:** Gastric cancer, Olfactomedin 4, RNA interference, Cell growth, Apoptosis resistance

## Abstract

**Background:**

Human olfactomedin 4 (OLFM4) gene is a secreted glycoprotein more commonly known as the anti-apoptotic molecule GW112. OLFM4 is found to be frequently up-regulated in many types of human tumors including gastric cancer and it was believed to play significant role in the progression of gastric cancer. Although the function of OLFM4 has been indicated in many studies, recent evidence strongly suggests a cell or tissue type-dependent role of OLFM4 in cell growth and apoptosis. The aim of this study is to examine the role of gastric cancer-specific expression of OLFM4 in cell growth and apoptosis resistance.

**Methods:**

OLFM4 expression was eliminated by RNA interference in SGC-7901 and MKN45 cells. Cell proliferation, anchorage-independent growth, cell cycle and apoptosis were characterized in vitro. Tumorigenicity was analyzed in vivo. The apoptosis and caspase-3 activation in response to hydrogen peroxide (H_2_O_2_) or tumor necrosis factor-alpha (TNF α) were assessed in the presence or absence of caspase inhibitor Z-VAD-fmk.

**Results:**

The elimination of OLFM4 protein by RNA interference in SGC-7901 and MKN45 cells significantly inhibits tumorigenicity both in vitro and in vivo by induction of cell G1 arrest (all P < 0.01). OLFM4 knockdown did not trigger obvious cell apoptosis but increased H_2_O_2 _or TNF α-induced apoptosis and caspase-3 activity (all P < 0.01). Treatment of Z-VAD-fmk attenuated caspase-3 activity and significantly reversed the H_2_O_2 _or TNF α-induced apoptosis in OLFM4 knockdown cells (all P < 0.01).

**Conclusion:**

Our study suggests that depletion of OLFM4 significantly inhibits tumorigenicity of the gastric cancer SGC-7901 and MKN45 cells. Blocking OLFM4 expression can sensitize gastric cancer cells to H_2_O_2 _or TNF α treatment by increasing caspase-3 dependent apoptosis. A combination strategy based on OLFM4 inhibition and anticancer drugs treatment may provide therapeutic potential in gastric cancer intervention.

## Background

Human OLFM4 (olfactomedin 4, also known as hGC-1, GW112), originally termed human cloned from myeloid precursor cells after granulocyte colony-stimulating factor stimulation [1], is a secreted glycoprotein more commonly known as the anti-apoptotic molecule GW112 [2,3]. OLFM4 is normally expressed in bone marrow, prostate, small intestine, stomach, colon and pancreas [1,4]. Subsequently, increased OLFM4 levels were also found in the crypt epithelium of inflamed colonic mucosa of inflammatory bowel diseases [5] and in gastric biopsies infected with Helicobacter pylori [6,7]. More recently, up-regulated OLFM4 expression has been described in lung and breast [8], prostatic [3], gastric [3,9] and pancreatic cancers [8,9] as well as in colorectal adenomas [10-14].

It has been suggested that OLFM4 is involved in cellular process such as apoptosis and tumor growth [2]. Although the cellular function of OLFM4 has been investigated, these results do not always coincident. Overexpression of OLFM4 has been shown to facilitate mouse prostate tumor Tramp-C1 cells growth in syngeneic C57/BL6 mice [2] but inhibit human prostate cancer PC-3 cell proliferation [15]. Moreover, up-regulated OLFM4 showed a strong anti-apoptotic activity in mouse lymphoid vein endothelial SVEC cells and human adenocarcinoma HeLa cells [1,2], whereas recent findings suggested a proapoptotic effect of OLFM4 in human myeloid leukemia HL-60 cells [16]. Evidence from these studies strongly suggests that roles of OLFM4 in cell growth control and apoptosis may depend on the cell or tissue type [10,13-15]. To date, however, very limited data concerning the role of OLFM4 in the cell growth and apoptosis profiles of gastric cancer cells has been published.

In the present study, we analyzed OLFM4 protein expression in gastric cancer cells and normal human gastric epithelial GES-1 cells by western blotting. Using plasmid-mediated short hairpin RNA (shRNA), we inhibited OLFM4 expression in the gastric cancer SGC-7901 and MKN45 cells to observe cell proliferation, cell cycle phase, apoptosis in vitro and to assess its tumorigenic capacity in vivo. We also explored the apoptosis and caspase-3 activation in response to cytotoxic agents such as H_2_O_2 _or TNF α in the presence or absence of caspase inhibitor Z-VAD-fmk between OLFM4 knockdown cells and HK control cells.

## Methods

### Cell culture, reagents and mice

The human gastric cancer cells BGC-823, HGC-27, SGC-7901, MKN28, MKN45 and human normal gastric epithelial GES-1 cells were maintained DMEM medium (GibcoBRL, Gaithersburg, MD) containing 10% fetal bovine serum (FBS, GibcoBRL, USA),100 U/ml of penicillin and 100 μg/ml of streptomycin. H_2_O_2 _and TNF-α were obtained from Sigma (St. Louis, MO) and Z-VAD-fmk was purchased from Calbiochem (San Diego, CA). BALB/C nude (nu/nu) mice (4-6 weeks old, SPF degree, 20 ± 3 g) were purchased from Laboratory Animal Center of Chongqing medical University (Chongqing, China). All procedures were conducted according to the internationally accepted ethical guidelines (NIH publication no. 85-23, revised 1985).

### Plasmid constructs and stable transfection

shRNA-mediated RNAi plasmid (pGenesil 1.1-siOLFM4) and a scrambled control plasmid (pGenesil 1.1-HK) were constructed to knock down the endogenous OLFM4 in SGC-7901 and MKN45 cells. After transfection and neomycin (G418) selection, OLFM4 knock-down SGC-7901-siOLFM4, MKN45-siOLFM4 cells and scrambled SGC-7901-HK, MKN45-HK control cells were stably obtained, respectively (details shown in Additional file 1: Supplementary data).

### RNA extraction and quantitative RT-PCR (qRT-PCR)

Total RNA in various cells or tumor xenografts was extracted using the RNeasy Mini Kit (Qiagen, CA, USA), and was followed by cDNA synthesis using the ReverTra Ace-α-first strand cDNA synthesis system (Toyobo, Osaka, Japan) as previous described [17]. Quantitative real-time PCR was performed using 7500 real-time PCR system (Applied Biosystems) with SYBR-Green as a fluorescent dye (Toyobo, Osaka, Japan) (details shown in Additional file 1: Supplementary data). Fold changes in gene expression were determined using the "2 - ddCT" method [18].

### Cell proliferation assay in vitro and cell viability measurement

Cell proliferation and cell viability were measured using Cell proliferation WST-1 kit (Roche) according to the manufacturer's instructions. For cell proliferation, cells were seeded at a density of 1 × 10^3 ^cells per well of a 96-well plates and grown for 5 days. The optical density (450 nm) value was detected using the Microplate Reader (Tacan, Swaziland) per day. Each assay was performed in triplicate. As for measurement of cell viability, cells (1 × 10^4^/well) were seeded at 200 μl of media in 96-well plates. After 12 h incubation, H_2_O_2 _or TNF α was treated in indicated concentrations. Relative absorbance was measured as described in cell proliferation.

### Anchorage-independent growth assay

Anchorage-independent growth was performed on soft agar to reflect in vitro clonogenicity. Briefly, cells (5 × 10^2^) from each colony were suspended in 0.3% agar in DMEM and then plated on solidified agar (0.5%) in 6-well dishes. Cells were incubated for 2 weeks at 37°C in 5% CO_2 _before the colonies was measured. Number of colonies was counted at 200 × magnification for five random fields. Each assay was performed in triplicate.

### Flow cytometry analysis

Flow cytometry (FCM) analysis was performed to assess cell cycle progression or apoptosis. (details shown in Additional file 1: Supplementary data)

### Caspase assay

Enzymatic activity of caspase-3 and -9 was measured using a fluorometric assay according to a method described previously [8].

### Western blot analysis

Western blotting was performed as described previously [17]. The following antibodies were used for western blotting: anti-OLFM4 (Abcam, Cambridge, UK) and anti-β-actin (Santa Cruz, CA, USA) The relative quantity of proteins was analyzed using Quantity One software (Bio-Rad, Hercules, CA, USA) and normalized to that of β-actin (details shown in Additional file 1: Supplementary data).

### Xenograft tumor model

Fourty nude mice were divided into four groups randomly. Each group was injected subcutaneously in the backs with the suspension of 200 μl containing 2 × 10^6 ^cells above mentioned, respectively. The volume of xenografts was serially measured. The mice were sacrificed after 35 days. The xenografts were excised and weighed. The inhibition rates of xenografts were calculated according to the formula: inhibition rate (%) = 1-mean weight (OLFM4 knock down cells-injected group or HK control cells-injected group)/mean weight (HK control cells-injected group) × 100%. Then, the tumor tissue was subject to total RNA isolation or immunohistochemistry detection.

### Immunohistochemistry (IHC)

OLFM4 proteins in tumor xenografts were analyzed by IHC using rabbit-anti OLFM4 (Abcam, Cambridge, UK) (details shown in Additional file 1: Supplementary data).

### Statistics

Data from independent experiments were expressed as the mean ± S.D. of at least three experiments. Comparisons between groups were analyzed by two-tailed Student's *t*-test or ANOVA, as appropriate, and p values < 0.05 were considered to be statistically significant.

## Results

### Efficient knock down of OLFM4 gene by plasmid-mediated siRNA in gastric cancer cells

**Figure 1:**
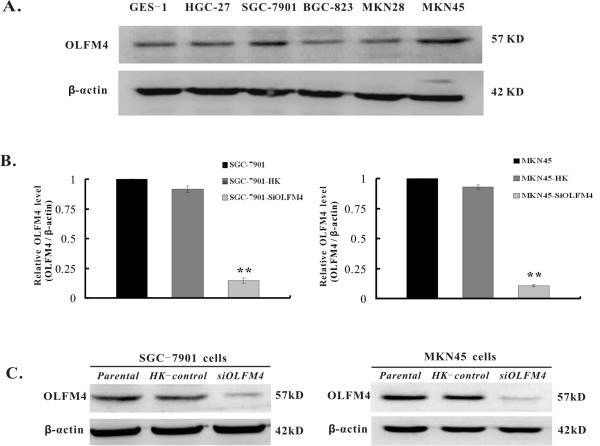
**Efficient knockdown of OLFM4 by RNA interference in gastric cancer SGC-7901 and MKN45 cells**. **A**. Expression of OLFM4 protein in various gastric cancer cell lines. Expression profile of OLFM4 protein was analyzed using western blotting with β-actin as an internal control. GES-1 cells were served as normal control cells. **B**. OLFM4 mRNA expression in OLFM4 knockdown cells. Relative expression of OLFM4 was detected by qRT-PCR. β-actin was used as an internal control. Fold changes in OLFM4 mRNA expression were determined using the 2^-ΔΔCt ^method. **C**. OLFM4 protein expression in OLFM4 knockdown cells. OLFM4 protein expression was detected by western blotting. β-actin protein was used as an internal control. Representatives of three experiments are shown. **P < 0.01 vs. HK control cells group (n = 3).

OLFM4 protein expression pattern was investigated in gastric cancer BGC-823, HGC-27, SGC-7901, MKN28, MKN45 cells and normal GES-1 control cells (Figure 1A). OLFM4 protein definitely expresses in all these gastric cancer cells and GES-1 cells. In particular, SGC-7901 and MKN45 cells expressed relative high level OLFM4 protein than other gastric cancer cells and GES-1 cells. Therefore, SGC-7901 and MKN45 cells were chosen for further studies.

To down-regulate OLFM4 expression, a plasmid-mediated shRNA targeting OLFM4 gene was constructed to stably knock down OLFM4 expression in SGC-7901 and MKN45 cells. As shown in Figure 1B, OLFM4 mRNA levels were significantly reduced in SGC-7901-siOLFM4 and MKN45-siOLFM4 cells compared to their HK control or parental cells (P < 0.01). These results were further confirmed by western blotting analysis (Figure 1C). No significant difference in OLFM4 expression was observed between parental and HK control cells. The levels of mRNA and protein for the β-actin were similar among the different groups. These results suggest that a plasmid-mediated OLFM4-siRNA can specifically and efficiently knock down OLFM4 level in gastric cancer cells. Given the analysis stated above, therefore, OLFM4 knock down cells and HK control cells were chosen for further investigation.

### OLFM4 knockdown inhibits gastric cancer cell proliferation and anchorage-independent growth in vitro

**Figure 2:**
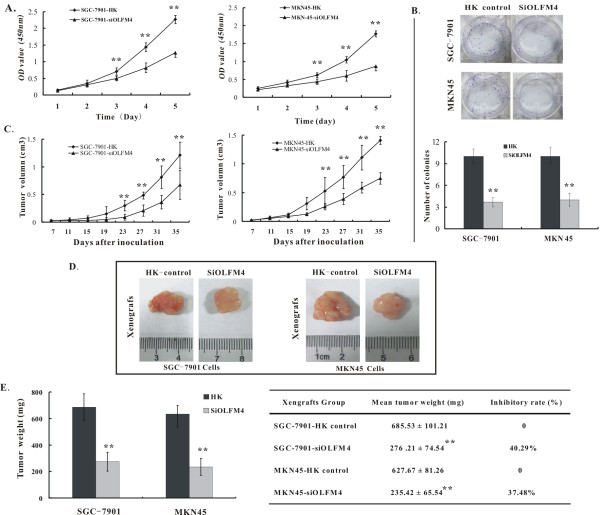
**knockdown of OLFM4 inhibits the growth of SGC-7901 and MKN45 cells in vitro and in vivo**. **A**. Cell growth curve. Cell proliferation in vitro was assessed by cell growth curve, as determined by counting the cell number (WST-1 assay) in the SGC-7901 cells (left panel) and MKN45 cells (right panel). The OD value (450 nm) was counted on the indicated days and presented as the mean cell numbers (n = 3). **B**. Anchorage-independent growth in soft agar. Representative images of three experiments were shown (upper panel). Data represent the mean number of colonies counted at 200 × magnification for 5 random fields (lower panel). **C**. The mean tumor volume after subcutaneous injection of nude mice with HK control or OLFM4 knockdown cells was measured at the indicated time points. **D**. Representative tumors images at 35 days after subcutaneous injection of indicated cells. **E**. Mean tumor weight (left panel) and inhibitory rate (right panel) in tumor xenografts. Data represent the mean tumor weight of xenografts (mean ± SD, n = 10). **P < 0.01 vs. HK control group.

To determine the role of OLFM4 in gastric cancer cell growth, we investigated the effect of OLFM4-siRNA on the cell growth at 1-5 day time point by WST-1 assay. As shown in Figure 2A, in all two gastric cancer cell lines, the growth of OLFM4 knock down cells was significantly reduced compared with HK control cells from day 3 (P < 0.01). To further examine the importance of OLFM4 in the tumorigenesis of gastric cancer cells in vitro, we carried out anchorage-independent growth assays and found SGC-7901 and MKN45 cells expressing HK controls grew well in soft agar, forming distinct colonies. In contrast, OLFM4 knockdown SGC-7901 and MKN45 cells exhibited a dramatic reduction in the number of soft agar colonies (P < 0.01) (Figure 2B), showing transforming abilities less than those of the control cells. These data indicated that knock-down of OLFM4 could inhibit gastric cancer cell proliferation and anchorage-independent growth in vitro.

### Growth-inhibitory effect of decreased OLFM4 in gastric tumor xenografts

Moreover, we also performed subcutaneous tumor formative assay in nude mice to evaluate the growth suppression effect of down-regulated OLFM4 in vivo. Nude mice were subcutaneously injected with OLFM4 knockdown or HK control cells. Tumor volumes per 4 days and tumor weight at 5 weeks were measured respectively after subcutaneous injection. As shown in Figure 2C-D, whether tumor volume or tumor weight produced by OLFM4 knock down SGC-7901 and MKN45 cells had significantly reduced growth compared with tumors produced in mice injected with HK control-transfected cells (P < 0.01). The inhibitory rate of SGC-7901 and MKN45 knock down cells-injected group on tumor growth was 40.29% and 37.48% respectively (Figure 2E).

**Figure 3:**
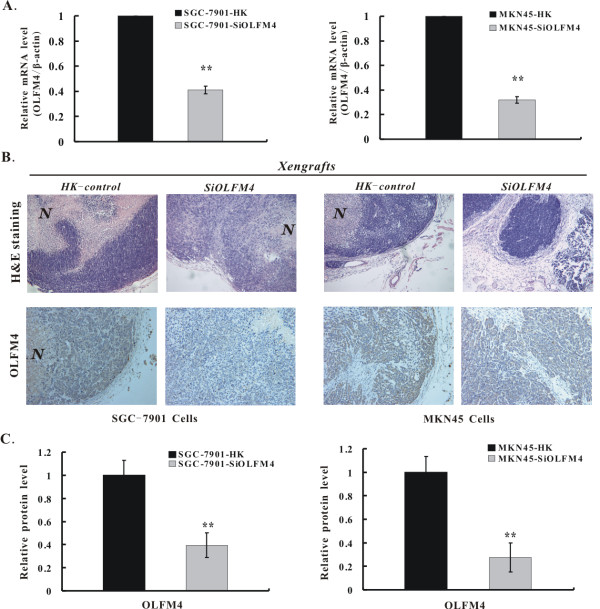
**qRT-PCR and IHC detection of OLFM4 in tumor xenografts**. **A**. qRT-PCR for OLFM4 mRNA in tumor xenografts. Fold changes in OLFM4 mRNA were determined using the 2^-ΔΔCt ^method. β-actin gene was used as an internal control. **B**. H&E staining (upper panel) and IHC for OLFM4 protein (lower panel) in tumor xenografts. Representative images (200×) are shown. N, Necrosis. **C**. Quantification analysis of OLFM4 expression. The average value was measured from five randomly different fields under the microscope (400×) using the Image-Pro PLUS V6.0. Data were expressed as mean ± SD. **P < 0.01 vs. HK control group.

To further assure OLFM4 expression is indeedly silenced after subcutaneous injection of nude mice, we also evaluated OLFM4 expression in tumor xenografts using qRT-PCR and IHC. Similarly in vitro, OLFM4 mRNA level in tumor xenografts produced by OLFM4 knockdown cells also showed a significant decrease compared with HK control tumors xenografts (P < 0.01) (Figure 3A). Consistent with the results of qRT-PCR, significant reduction of OLFM4 protein was also observed in tumor xenografts by IHC (P < 0.01) (Figure 3B and 3C). Higher gray scale and stronger positive signal by DAB visualization were found in tumor xenografts of HK control cells-injected group. On the contrary, weak brown staining was observed in tumor xenografts of OLFM4 knockdown cells-injected group. Additionally, more large necrosis region was observed in tumor xenografts produced by HK control cells due to a fast growth of HK control cells (Figure 3B upper panel). These data provided a strong indication that OLFM4 expression at mRNA and protein levels are stably inhibited indeed in vivo. Taken collectively, both in vitro and in vivo experiments suggest that OLFM4 knockdown inhibits the growth of gastric cancer cells.

### OLFM4 knock-down delays G1 to S transition but does not trigger obvious apoptosis in gastric cancer cells

**Figure 4:**
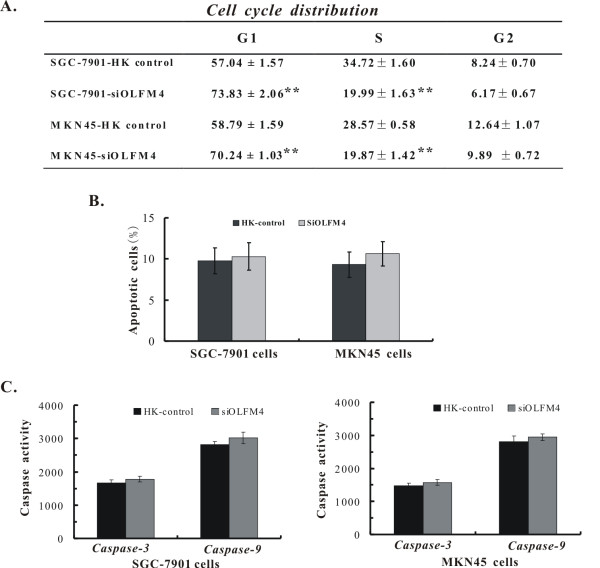
**Assessment of cell cycle distribution, apoptotic cells and caspase-3/9 activation in OLFM4 knock down gastric cancer cells**. **A**. Cell cycle analysis. Changes in cell cycle distribution of SGC-7901-HK, SGC-7901-siOLFM4 and MKN45-HK, MKN45-siOLFM4 cells were analyzed by FCM. Data represent the mean percentage of cell-cycle phase distribution (n = 3). **B**. Cell apoptosis analysis. Apoptosis was estimated with AnnexinV-PE/7-AAD staining by FCM. Data represent the mean apoptotic cell percentage (n = 3). **C**. Caspase-3 and -9 activations. Caspase-3, 9 activations between indicated cells are shown. **P < 0.01 vs. HK control group.

Given our observed inhibitory effects on cell growth in vitro and in vivo, we sought to determine whether enhanced apoptosis or delayed cell cycle progression was associated with growth inhibition. We first evaluated the effect of decreased OLFM4 on cell cycle progression. As shown in Figure 4A, both OLFM4 knockdown SGC-7901 and MKN45 cells showed significant increased numbers in G1 phase and decreased numbers in S phase, in contrast to their HK control cells (P < 0.01). No significant differences were observed in the G2/M-phase, indicating a typical G1 delay of cell cycle.

To determine whether apoptosis is involved in this growth inhibition, we next performed cell apoptosis analysis. Interestingly, reduced OLFM4 expression could not result in significant changes in apoptosis (Figure 4B), which is consistent with the cell cycle analysis showing no apparent sub-G1 phase in the tested cells. To further examine alterations of apoptotic signals, caspase-3/-9 activity was also identified by colorimetric activation assays. Both caspase-3 and -9 activations showed no significant changes after knockdown of OLFM4 in SGC-7901 and MKN45 cells (Figure 4C). These results suggest that down-regulation of OLFM4 may exert an inhibitory effect on cell growth by regulating cell cycle progression not involving apoptosis in gastric cancer cells.

### Deletion of OLFM4 sensitizes gastric cancer cells to H_2_O_2 _or TNF α-induced apoptosis

**Figure 5:**
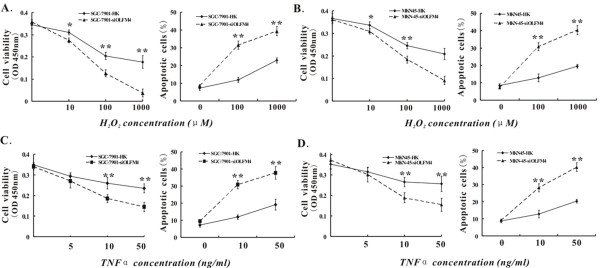
**Response of OLFM4 knockdown SGC-7901 and MKN45 cells to apoptosis-inducing agents H_2_O_2 _or TNF α**. OLFM4 knockdown and HK control cells were treated with H_2_O_2 _(**A **and **B**) or TNF α (**C **and **D**) as indicated doses for 12 h. Cell viability was measured by WST-1 assay (**A-D **left panels) and expressed in the mean OD value (450 nm) (n = 3). The percentage of the apoptotic cells was determined by FCM (**A-D **right panels) and expressed in the mean apoptotic percentage (n = 3). *P < 0.05,**P < 0.01 vs. HK control group.

The distinct effect of H_2_O_2 _or TNF α on the apoptosis between OLFM4 knock down and HK control cells was also investigated. OLFM4 knock down or HK control cells were treated with 10, 100 and 1000 μM H_2_O_2 _or 5, 10 and 50 ng/ml TNF α respectively. Cell viability and apoptosis were assessed. As shown in Figure 5A-D, a dose-dependent decrease in cell viability was observed in H_2_O_2 _or TNF α-treated OLFM4 knock down cells. Furthermore, treatment of 10 μM H_2_O_2 _(Figure 5A-B) or 10 ng/ml TNF α (Figure 5C-D) led to a significant reduction in cell viability in OLFM4 knock down cells compared with HK control cells (P < 0.01). Of particular interest is the finding that OLFM4 knockdown cells rather than HK control cells treated with 10~1000 μM H_2_O_2 _exhibited more prominent apoptotic percentages in comparison to those treated with PBS mock (P < 0.01) (Figure 5A-B). Similar results were also seen in TNF α-treated OLFM4 knockdown cells (Figure 5C-D). In other words, OLFM4 knock down enhanced H_2_O_2 _or TNF α-induced apoptosis in gastric cancer cells, indicating deletion of OLFM4 made SGC-7901 and MKN45 cells more sensitive to treatment of H_2_O_2 _or TNF α.

### Caspase-3 activity is involved in H_2_O_2 _or TNF α-induced apoptosis in OLFM4 knock-down cells

**Figure 6:**
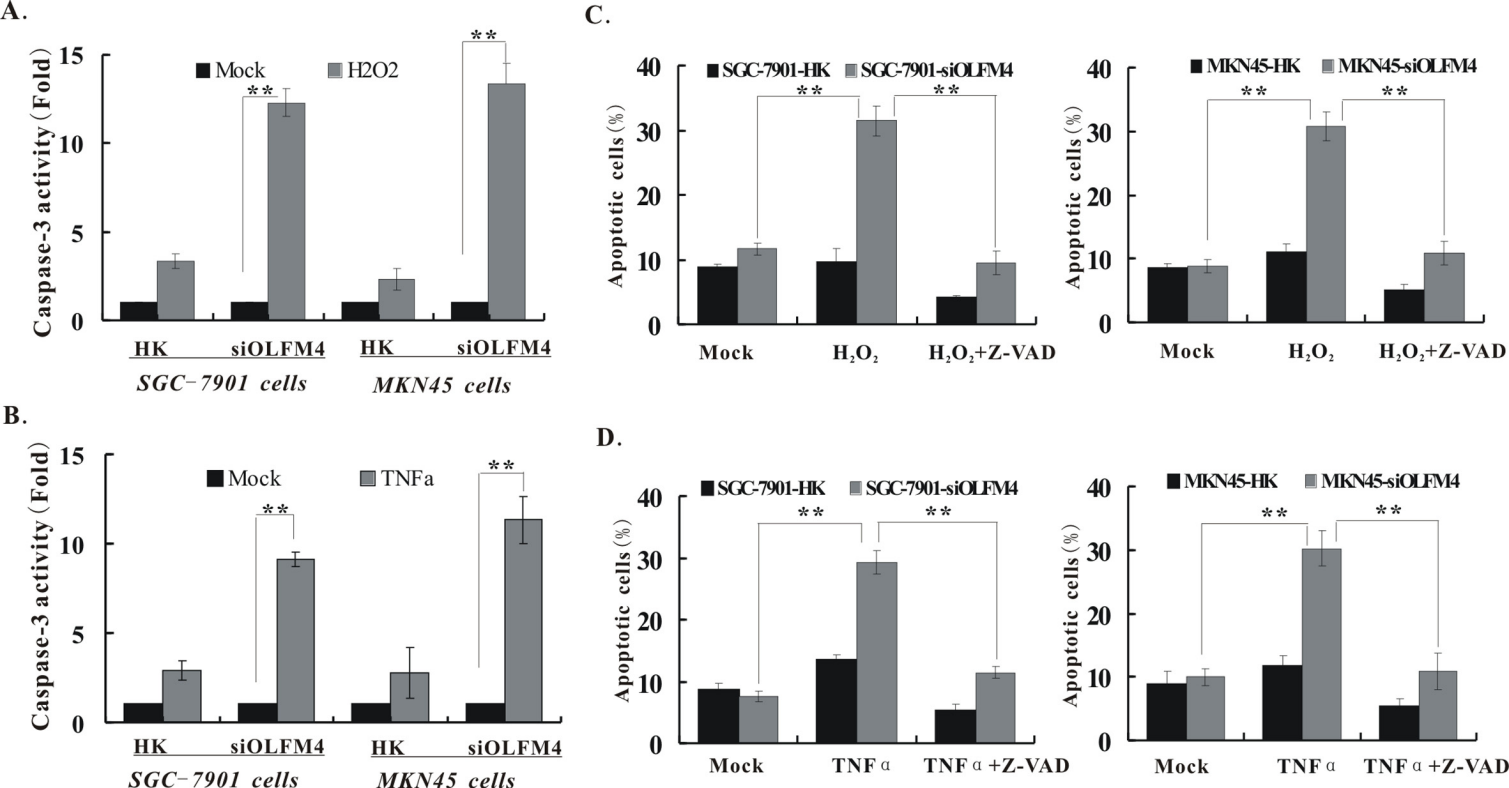
**Involvement of caspase-3 activity in H_2_O_2 _or TNF α-induced apoptosis in OLFM4 knockdown SGC-7901 and MKN45 cells**. **(A-B) **Increased caspase-3 activity in H_2_O_2 _or TNF α-treated OLFM4 knockdown cells. OLFM4 knockdown and HK-control cells were treated with 10 μM H_2_O_2 _**(A) **or 10 ng/ml TNF α **(B) **for 12 h. Caspase-3 activity was measured using colorimetric assay and expressed in fold changes. **P < 0.01 versus PBS mock group (n = 3). **(C-D) **Reversed cell apoptosis by caspase inhibitor in OLFM4 knockdown cells. Cells were treated with H_2_O_2 _or TNF α alone with indicated doses as above mentioned or pretreated with 20 μM Z-VAD-fmk 2 h before H_2_O_2 _or TNF α treatment. The percentage of apoptotic cells was determined by FCM. Data represent the means ± SD from three independent experiments. ** p < 0.01 vs. H_2_O_2 _**(C) **or TNF α **(D)**-treated OLFM4 knockdown cells group.

The above results indicated that knock down of OLFM4 could increase H_2_O_2 _or TNF α-stimulated apoptosis. To further verify whether caspase-3 is activated in the H_2_O_2 _or TNF α-induced apoptosis in OLFM4 knockdown cells, we next detected caspase-3 activation in H_2_O_2 _or TNF α-treated cells using colorimetric assay. As shown in Figure 6A, treatment of H_2_O_2 _or TNF α resulted in much more enhancement of caspase-3 activity in OLFM4 knockdown cells than HK control cells (P < 0.01), suggesting caspase-3 activity is involved in the H_2_O_2 _or TNF α-induced apoptosis in OLFM4 knockdown cells. To further verify whether H_2_O_2 _or TNF α-induced apoptosis is dependent on caspase-3 activity, Z-VAD-fmk, a caspase inhibitor was used before the treatment of H_2_O_2 _or TNF α. Pre-treatment of Z-VAD-fmk significantly attenuated H_2_O_2 _or TNF α-induced cell apoptosis as well as caspase-3 activity in both OLFM4 knockdown cells (P < 0.01) (Figure 6C-D), indicating that H_2_O_2 _or TNF α-induced apoptosis is caspase-3 dependent in OLFM4 knockdown cells.

## Discussion

Recently accumulating data demonstrated OLFM4 is frequently overexpressed in many types of human tumors including gastric cancer, and it was believed to play a crucial role in the development and progression of gastric carcinogenesis [19,20]. Although previous studies have shown that OLFM4 is involved in apoptosis and tumor growth, recent observations also suggest that cell or tissue-specific effects may exist for the OLFM4 gene. Relatively little is known regarding the tumor growth and apoptosis underlying gastric cancer-specific OLFM4 expression. To gain a better understanding of this role for the altered OLFM4 in human gastric cancer, experimental support is required to validate the role of the OLFM4 gene in gastric cancer.

It has been shown that abnormal expression of hGC-1 may be regulated at the transcriptional or posttranscriptional level [13]. In our present works, we directly investigated OLFM4 protein expression pattern in gastric cancer cells and normal GES-1 cells. SGC-7901 and MKN45 cells expressing relative high OLFM4 levels were chosen for this study. Since reducing the target gene expression by genetic means in established cell lines is helpful for a better understanding of its role in maintaining the malignant phenotype particularly in analyzing genes that are essential for cellular survival [21], we generated stable clone pools of SGC-7901 and MKN45 expressing OLFM4-siRNA or scrambled HK control by the plasmid-based siRNA approach and confirmed knock-down efficiency of OLFM4 gene at mRNA and protein levels by qRT-PCR and western blotting.

Our present works demonstrate that OLFM4 plays an essential role in gastric cancer tumorigenesis. Knockdown of OLFM4 inhibits cell proliferation and anchorage-independent growth ability in vitro. Xenograft tumor model in vivo also implies that decreased OLFM4 can inhibit the tumor growth of human gastric cancer cells. These results indicate that OLFM4 plays a crucial role in cell proliferation of gastric cancer cells. Our results also observed that knock-down of OLFM4 did not influence the rate of apoptosis and caspase-3 and 9 activations in OLFM4 knockdown cells, suggesting that apoptosis might not be the mechanism underlying the inhibition of tumor growth. Thus, we postulate that OLFM4 expression is not essential for cancer cell survival, which is in accordance with a recent observation that genetic knock-out mice for OLFM4 show normal development and hematopoietic phenotypes [17]. To further characterize the mechanism underlying growth inhibition, we performed cell cycle analysis and demonstrated that inhibition of OLFM4 expression induced gastric cancer cells to accumulate in G1 phase of the cell cycle, suggesting that down-regulated OLFM4 may exert an inhibitory effect on cell growth by a mechanism regulating cell cycle progression not involving apoptosis in gastric cancer cells.

Resistance of tumor cells to the induction of apoptosis is one of the main factors responsible for the failure of many conventional anticancer therapies that use anticancer agents and radiation. Therefore, apoptosis control in cancer cells is of critical biological and clinical importance [22,23]. Anti-apoptotic activity is another important function of OLFM4 gene [2]. In particular, H_2_O_2_-induced cellular apoptosis was attenuated by overexpressed OLFM4 in a prostatic cancer cell line [2]. Moreover, MKN45 cells have been shown an ability of resistance to TNF α-induced apoptosis [24]. Given these findings, we hypothesize that knockdown of OLFM4 expression might enhance H_2_O_2 _or TNF α-induced apoptosis in gastric cancer cells. Here, we showed that knock-down of OLFM4 effectively enhanced cell apoptosis in response to H_2_O_2 _or TNF α stimulation in MKN45 as well as SGC-7901 cells, suggesting blocking OLFM4 may increase the susceptibility of gastric cancer cells to the presence of H_2_O_2 _or TNF α.

As it is well known that caspase-3, a key executive molecule of the apoptotic pathway, plays a critical role in apoptotic processes in a variety of cells. We further examined caspase-3 activation in OLFM4 knockdown and HK control cells in the presence or absence of caspase inhibitor Z-VAD-fmk. We observed that treatment of H_2_O_2 _or TNF α significantly up-regulated Caspase-3 activity in OLFM4 knockdown cells than HK control cells while pre-treatment with Z-VAD-fmk reversed caspase-3 activity as well as H_2_O_2 _or TNF α-induced apoptosis. The results indicate that H_2_O_2 _or TNF α-induced apoptosis in OLFM4 knockdown cells is caspase-3 dependent. Based on the present data, it is conceivable that up-regulated OLFM4 enables gastric cancer cells to resist apoptosis induction by decreasing the susceptibility to anticancer drugs.

In fact, antagonists of OLFM4 have been reported to inhibit the proliferation in others types of cancer cells such as human pancreatic cancer PANC-1 cells [8] and human lung caner SBC-1 cells [9]. However, controversial results have also been observed. Decreased OLFM4 mRNA inhibit PANC-1 cells proliferation by S to G2/M phase arrest [8], which is different from our results showing delayed G1 phase progress in gastric cancer. Certain important details (or reasons) might explain this discrepancy. First, as recent studies suggested, a cell or tissue specific role of OLFM4 (gastric cancer cells and pancreatic cancer cells) may be a persuasive explanation for this discrepancy. The most noteworthy is, OLFM4 expression at mRNA level but not protein level in PANC-1 cells was successfully measured using RT-PCR [8] whereas the most recent report by Kim et al. showed that PANC-1 cells has no OLFM4 mRNA expression [25], indicating the expression pattern and role of OLFM4 gene in PANC-1 cells need further confirmation.

Despite OLFM4 silencing was shown to an inhibitory effect on cell growth and a decreased resistance to H_2_O_2 _or TNF α treatment in gastric cancer cells, it is not eliminated that other mechanisms may also be regulated by OLFM4 and contributes to growth inhibition and apoptosis control signaling, considering the crosstalk of the network. Indeed, OLFM4, a target gene of NF-κB pathway [16,17,25], has also shown a negative feedback effect on *H. pylori *infection-induced NF-κB activation in HEK 293 T cells [6], indicating the regulatory pathways controlled by OLFM4 in gastric cancer could be involved in a very complex and intricate network. Therefore, complex interactions between OLFM4 and other signaling intermediates are needed to be more extensive investigation in our future studies.

## Conclusions

Taken together, the present study provides evidences that the elimination of OLFM4 expression in gastric cancer SGC-7901 and MKN45 cells inhibits tumorigenicity both in vitro and in vivo by regulating cell cycle progression not involving apoptosis. Moreover, suppression of OLFM4 enhances caspase-3 dependent apoptosis in response to H_2_O_2 _or TNF α in human gastric cancer cells. By understanding the role of OLFM4 in tumor growth and apoptosis resistance, it may be possible to develop a perspective strategy based on a combination of OLFM4 inhibition and anticancer drugs treatment in gastric cancer intervention.

## Abbreviations

OLFM4: Olfactomedin 4; H_2_O_2_: Hydrogen peroxide; IHC: Immunohistochemistry; TNF α: Tumor necrosis factor-alpha; shRNA: Short hairpin RNA; RNAi: RNA interference.

## Competing interests

The authors declare that they have no competing interests.

## Authors' contributions

RHL, MHY performed the majority of the experiments, contributed to the experimental design, and drafted the manuscript. HX, LMB and HAY contributed to experimental design and data discussion. LWY contributed to statistical data analysis. XJ, NA, LYW assisted in the writing of and proofed the manuscript. YH designed and supervised the experiments. All authors read and approved the final draft of the manuscript.

## Supplementary Material

Additional file 1 **Supplementary data**. Click here for file
