# Supplementary material for: Depletion of OLFM4 gene inhibits cell growth and increases sensitization to hydrogen peroxide and tumor necrosis factor-alpha induced-apoptosis in gastric cancer cells
Source: J Biomed Sci. 2012 Apr 3;19(1):38. doi: 10.1186/1423-0127-19-38 (PMC3359197; doi:10.1186/1423-0127-19-38)
Supplement: Additional file 1 — Supplementary data. [file 1423-0127-19-38-S1.DOC]

**Supplementary data**

**Material and methods**

*1. Plasmid construction and transfection*

*1.1.OLFM4-siRNA expression vectors and negative control*

As previously described [1,2], nucleotides 299-319 of OLFM4 (accession number: NM_006418.3) , annealed oligonucleotides (listed below) were cloned into siRNA expression vector-pGenesil-1.1 (Genesil Biotechnology Corp. Wuhan, China), in which expression of a gene is under the control of the RNA polymerase-Ш U6-RNA gene promoter. The oligonucleotides were as follows: sense, 5′-CACCAACGCTTGGAATTCACAGC TCTTCAAGACGGAGCTGTGAATTCCAAGCGTTTTTTTTG-3′ and antisense, 5′-AGCTCAAAAAAAACGCTTGGAATTCACAGCTCCGTCTTGAAG AGCTGTG A ATTCCAAGCGTT-3′. The resulting siRNA expression was further verified by DNA sequencing and was named pGenesil 1.1-siOLFM4. As for control, a common commercial plasmids pGenesil 1.1-HK (Genesil Biotechnology Corp) was used for negative control.

*1.2. Transfection*

SGC-7901 and MKN45 cells were grown in normal culture media to a 50% confluent state and were then transfected with pGenesil 1.1-siOLFM4 and pGenesil 1.1-HK control using Fugene HD reagent (Roche, OH, USA). Twenty-four hours after transfection, cells were maintained in the growth medium containing neomycin G418 (600ug/ml) (Amersco, OH, USA) for 4 weeks to generate stable OLFM4 knock down cell clones (SGC-7901-siOLFM4 and MKN45-siOLFM4) and stable scrambled HK control cell clones (SGC-7901-HK and MKN45-HK), respectively.

*2. Real time reverse transcription (RT)-PCR*

cDNA was synthesized from approximately 2 μg of total RNA using ReverTra-Ace reverse transcriptase (Toyobo, Osaka, Japan) and random primer 9 for 1 h at 37°C. For quantitative real-time PCR, the ABI PRISM 7500 Sequence Detection System (Applied Biosystems, Foster City, CA) and SYBR Green PCR Master Mix (Toyobo, Osaka, Japan) were used with specific primers as follows: OLFM4: 5′-AGCTCTTTCCCAGGTGTTGA-3′ (forward), 5′-AAGCGTTCCACTCTGTCCAC-3′ (reverse); β-actin: 5′-CCAACCGCGAGAAGATGA-3′ (forward), 5′-CCAGAGGCGTACAGGGATAG-3′ (reverse).The specificity of products generated by each set of primers was examined using gel electrophoresis and further confirmed by a melting curve analysis. Quantification was carried out by normalizing levels to the amount of total cDNA using the ubiquitously expressed β-actin as a standard. Fold changes in gene expression were determined using the “2−ddCT” method.

*3. Western blot analysis*

Cells were washed thrice with ice-cold PBS (pH 7.4), and lysed with ice-cold strong RIPA buffer (Beyotime, haimen, China) containing Complete Protease inhibitor tablets (Roche, Mannheim, Germany). The lysate was clarified by centrifugation at 15,000×g for 30 minutes at 4 ºC. The protein content was determined by using the BCA protein assay kit (Pierce, Rockford, Illinois, USA) according to the manufacturer’s instructions. The same amount of protein was loaded in each lane, separated by 8–12% SDS-PAGE and then transferred to PVDF membranes. The membranes were blocked with 5% nonfat milk, and then the membrane-bound proteins were probed with primary antibodies : OLFM4 (Abcam, Cambridge, UK) or β-actin (Santa Cruz Biotechnology, CA, USA) followed by secondary horseradish peroxidase-conjugated antibodies. Protein bands were visualized by a supersignal chemiluminescence detection (ECL) kit (Pierce, Rockford, Illinois, USA).

*4. Flow Cytometry Analysis*

Flow cytometry (FCM) analysis was performed to assess cell cycle progression and apoptosis. The cells plated onto six-well culture dishes (Costar, Tokyo, Japan) were trypsinized, and washed with FBS free media and PBS buffer. The cells were treated with 1 mL hypotonic fluorochrome solution (50 μg/ml PI, 0.1% sodium cutrate, 0.1% Triton X-100) at room temperature for 30 min. The cells were then kept on ice and 20 000 cells/sample were analyzed using the FCM.

Apoptosis was quantified by the combined staining of Annexin-PE and 7-amino-actinomycin D (7’-AAD) using the Annexin-PE/7’-AAD apoptosis detection kit (Kaiji, Nanjing, China). In some cases, cells were treated with H_2_O_2_ or TNF α alone with indicated doses or pretreated with 20 μM Z-VAD-fmk (pancaspase inhibitor) 2 h before H_2_O_2_ or TNF α treatment. Briefly, cells were harvested and resuspended in 500 μl of 1×binding buffer. After adding 10 μl of the Annexin V-PE solution and 5 μl of the 7’-AAD solution, the cells were incubated for 15 min at room temperature in the dark. At the end of incubation, the cells were analyzed by FCM using an BD FACS Canto system (BD Biosciences, USA). All experiments were carried out at least three times for each experimental condition.

*5. Immunohistochemistry*

The tumor xenografts were embedded in paraffin and sectioned at 5 µm. After deparaffinization of the sections, antigen retrieval was done by boiling the samples in citrate buffer for 15 minutes at 92 ºC-98 ºC and trypsinizing with 0.125% trypsin for 15 minutes at 37 ºC. The tissues were treated with 30 g/l BSA in PBS for 20 min at 37 ºC to block non-specific staining, and then incubated with human rabbit-anti human OLFM4 antibody (1:200 dilution) (Abcam, Cambridge, UK) in PBS containing 30 g/L BSA at 4 ºC for 16 h, the sections were then incubated with goat anti-rabbit secondary antibodies (IgG/HRP) for 15 minutes at 37 ºC using Non-Bio Two-Step Histostain™ -Plus kits, DAB staining (Zhongshan Goldenbridge Biotechnology, Corp. Beijing, China). The nucleus was counterstained with hematoxylin. The rest of the procedures were performed in accordance with the manufacturer’s instructions. The immunohistologic staining for OLFM4 was observed by a light microscopy.

**References**

1. Koshida S, Kobayashi D, Moriai R, Tsuji N, Watanabe N: **Specific overexpression of OLFM4(GW112/HGC-1) mRNA in colon, breast and lung cancer tissues detected using quantitative analysis.** *Cancer Sci* 2007, **98**:315-320.
2. Kobayashi D, Koshida S, Moriai R, Tsuji N, Watanabe N: **Olfactomedin 4 promotes S-phase transition in proliferation of pancreatic cancer cells.** *Cancer Sci* 2007, **98**:334-340.
